# Genome-Wide Identification and Analysis of Variants in Domestic and Wild Bactrian Camels Using Whole-Genome Sequencing Data

**DOI:** 10.1155/2020/2430846

**Published:** 2020-07-15

**Authors:** Nemat Hedayat-Evrigh, Reza Khalkhali-Evrigh, Mohammad Reza Bakhtiarizadeh

**Affiliations:** ^1^Department of Animal Science, Faculty of Agriculture and Natural Resources, University of Mohaghegh Ardabili, Ardabil 5619911367, Iran; ^2^Department of Animal and Poultry Science, College of Aburaihan, University of Tehran, Tehran 3391653755, Iran

## Abstract

The population size of Bactrian camels is smaller than dromedary, and they are distributed in cold and mountain regions and are also at the risk of extinction in some countries such as Iran. To identify and investigate the genome-wide variations, whole-genome sequencing of two Iranian Bactrian camels were performed with 37.4- and 42.6-fold coverage for the first time. Along with Iranian Bactrian camels, sequencing data from two Mongolian domestic and two wild Bactrian camels deposited in the NCBI were reanalyzed. The analysis eventuated to the identification of 4,908,998, 4,485,725, and 4,706,654 SNPs for Iranian, Mongolian domestic, and wild Bactrian camels, respectively. Also, INDEL variations ranged from 358,311 to 533,188 in all six camels. Results of variants annotation in all samples revealed that more than 88 percent of SNPs and INDELs were located in the intergenic and intronic regions. We found that 800,530 SNPs were common among all studied camels, containing 4,046 missense variants that affected 2,428 genes. Investigation of common genes among all camels containing the missense SNPs showed that there are 98 zinc finger and 4 fertility-related genes (ZP1, ZP2, ZP4, and ZPBP) in this set.

## 1. Introduction

Since the Camelidae family has been evolved in North America during Eocene [1], its members have gone a long way to evolution to date. Migration of camel's ancestors in different paths divided them into the Old World (migrated to east) and New World camels (migrated to South America) [2]. The Old World camels include three species, *Camelus dromedarius* (dromedary camel or one-humped camel), *Camelus bactrianus* (Bactrian camel or two-humped camel), and *Camelus ferus* (wild Bactrian camel or wild two-humped camel). Dissimilar to dromedaries living in hot and dry deserts of northern and eastern Africa, the Arabian Peninsula, and southwest Asia [3], the Bactrian camels are distributed in the cold and mountainous regions of Iran, China, Russia, Mongolia, and Kazakhstan [4]. The wild Bactrian camel, known as the only wild survivor of Old World camels, is living in northwestern China and southwestern Mongolia, especially the Outer Altai Gobi Desert [5]. Bactrian population in Iran (less than 150 camels) is in alarming status [6] and a suitable strategy is needed to enhance the population situation. They have been distributed in the Ardabil province located in the northwest of Iran in the border of Azerbaijan.

Camel is the most important animal to provide food in desert areas where other animals are not capable to live and produce. According to FAO, hunger has increased in the world in recent years, so that 821 million people (one out of every nine people in the world) are undernourished [7]. Camels support a significant part of food security in arid and semi-arid regions of Africa and Asia [6]. The use of new technologies in domesticated animal breeding, as an important source of food for humans, will help us raise the quality and quantity of animal source foods and enhance food safety especially in areas with the harsh conditions.

Whole-genome sequencing (WGS), as an efficient technology in livestock breeding, provides an opportunity to the better understanding of the genomic structure of desired farm animals. WGS is capable of producing more complete structural genetic information on individuals in comparison with technologies such as SNP chips [8]. Also, genome-wide studies as a key tool in the biology era can expand our information about endangered species to monitor and management of their biodiversity [9] and for designing practical strategies to save them from extinction.

The main purpose of the present study was to identify the genome-wide variations (SNPs and INDELs) of Iranian Bactrian camels and to compare them with domestic and wild Bactrian camels from other geographical regions. Functional and regional classifications of identified variants and gene ontology (GO) analysis on affected genes by some classes of variants were among the other goals of this work.

## 2. Materials and Methods

### 2.1. Animals, Sampling, and Total DNA Extraction

Whole-genome sequencing of one 9- (Bac1) and one 11-year old (Bac2) female Bactrian camels was performed to variants identification, variants annotation, and gene ontology analysis of genes containing variants. Bactrian camels in Iran have some characteristics such as the neck, front legs, and humps covered with wool; also, shoulder height and body length in adult camels are 153 and 123 centimetres, respectively (Supplementary 2). All animal care and experiments were approved by the animal science committee of the University of Mohaghegh Ardabili, Iran. Also, all experiments were performed in accordance with a routine guideline which is acceptable by this committee.

Genomic DNA was extracted using an RBC mini kit for mammalian blood with respect to the manufacturer's protocols (Real Biotech; South Korea). The quality of the extracted DNA for each camel was monitored using gel electrophoresis in 1% agarose gel. One paired-end library comprising two lanes was constructed for each camel. The length of reads was 2 × 100 bp with an average insert size of ~365 and ~360 bp for Bac1 and Bac2, respectively. The sequencing of constructed libraries was carried out using the Illumina HiSeq 2000 system (Illumina, San Diego, CA). To compare our samples with other breeds of Bactrian camels, we downloaded publicly deposited sequenced short reads of two domestic (accessions: SRX973830 and SRX973833) as well as two wild Bactrian camels (accessions: SRX973840 and SRX973843) from SRA-NCBI (short reads archive).

### 2.2. Quality Control, Trimming, and Short Reads Mapping

FastQC (http://www.bioinformatics.bbsrc.ac.uk/projects/fastqc/) was used to perform a general overview of short reads quality status; in fact, this program allows us to get general information about our data and make better decisions for the downstream analysis. Trimmomatic v.0.36 (MaxInfo approach) [10] was applied to quality filtration of raw sequencing reads for all individuals. After quality filtration, the reads less than 40 bp in length were removed. Supplementary 2 presents the output of quality checking of one of the studied samples, before and after trimming.

For mapping the clean reads to reference genome (RefSeq assembly accession: GCF_000767855.1; Ca_bactrianus_MBC_1.0), BWA v.0.7.15 [11] was used.

### 2.3. Postalignment Processes and Variant Calling

In many large-scale sequencing projects, several processes are performed as postalignment processes between the mapping of reads to reference genome to variant calling for decreasing erroneous calls [12]. Marking duplication was performed using Picard (http://broadinstitute.github.io/picard), and GATK [13] was used for local realignment and base quality score recalibration. We used SAMtools [14] to call SNPs and INDELs for individuals and called variants filtered as described by Khalkhali-Evrigh et al. [15].

### 2.4. Annotation of Called Variants

Due to the flexibility of SnpEff [16] to annotate genomic variants of animals without a database, we selected it for annotation of identified variants in the present work. Similar to our previous project on dromedary camels [15], due to the lack of database for Bactrian camels among the prebuilt databases for SnpEff, we built a database for Bactrian camels using the Bactrian reference genome and its GFF file using the database building guidelines (http://snpeff.sourceforge.net/SnpEff_manual.html#databases) in SnpEff.

### 2.5. Gene Ontology (GO) Analysis of Genes Containing Variants

DAVID v.6.8 [17] was used to the functional enrichment analysis of genes containing SNPs and INDELs. For this purpose, we extracted the genes affected by missense SNPs from shared SNPs among all samples, shared only between Iranian Bactrian camels (only_B1B2), shared only between Mongolian domestic Bactrian camels (only_DC), and shared only between wild Bactrian camels (only_WC). Also genes affected by frameshift INDELs were classified using gene ontology analysis. After uploading desired genes in DAVID, due to incomplete information of camels in mentioned program, we selected cattle (*Bos taurus*) as species and background species. Cattle was chosen based on Wu et al.'s [18] study suggested that the estimated divergence time between camelids and cattle is closer compared to other mammalian. The calculated *p* values were corrected using the Benjamini correction for multiple testing, and enriched terms were considered statistically significant at *p* adjusted < 0.1.

## 3. Results

### 3.1. Massively Parallel Sequencing of the Iranian Bactrian Camels

Whole-genome sequencing of two female mature Bactrian camels sampled from the Jahad Abad station located in Meshkin-Shahr (Ardabil province) yielded 813,158,076 and 933,365,664 short reads of 100 bp for Bac1 and Bac2, respectively. After removing of error-prone and low-quality bases and reads from initial raw reads, approximately 98.09% (797,679,174 reads) and 97.66% (911,654,292 reads) were remained as clean reads for Bac1 and Bac2, respectively. The percentage of retained reads for downloaded samples was higher than our samples (Table 1). Approximately, 98.7 (for WC247) to 98.89% (for DC408) of clean reads that belonged to non-Iranian Bactrian camels were mapped to reference genome, while this percentage for Bac1 and Bac2 was 97.28 and 97.74%, respectively. Successfully mapped reads to reference genome that yielded coverage ranged from 30.1 for DC399 to 42.6-fold for Bac2. We estimated fold coverage by dividing the successfully mapped bases by the length of the used Bactrian reference genome for mapping (1,992,663,268 bp).

### 3.2. SNPs and INDELs

**Figure 1:**
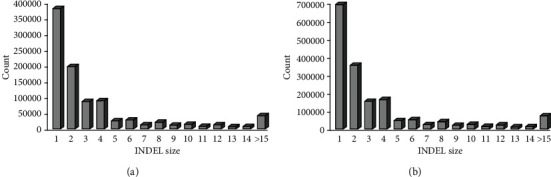
Length distribution of INDELs in genic (a) and nongenic (b) regions of all studied Bactrian camels.

Totally, 3,917,247 SNPs and 533,188 INDELs for Bac1 and also 3,677,474 SNPs and 481,305 INDELs for Bac2 were identified in the present work. Detected SNPs for non-Iranian camels ranged from 3,080,659 for DC399 to 3,656,164 for WC247 (Table 2). Similar to SNPs, identified INDELs for Mongolia domestic Bactrians (totally 752,523 INDELs) were less than wild Bactrian camels (totally 884,630 INDELs). It is worth pointing out that more than 51.57% of detected INDELs belonged to insertions for all studied camels. Variants with one and two base changes comprised more than 56 percent (ranged from 56.9% for Bac1 to 64.5% for WC305) of INDELs among all of the individuals. Investigation of length distribution for the located INDELs in genic regions (e.g., intron region) was carried out after the annotation of variants in comparison with nongenic regions (e.g., intergenic region). In terms of the length distribution pattern of INDELs, there was no significant difference between genic and nongenic regions (Figure 1). Also, the insertions include 53 percent of INDELs in both regions. It is worth noting that the lengths of INDELs were distributed from -45 for deletions to +30 for insertions.

**Figure 2:**
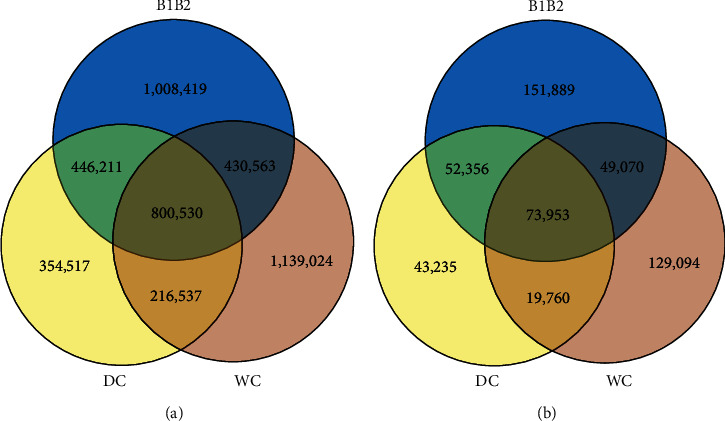
Venn diagram showing the overlap of SNPs (a) and INDELs (b) for common variants between Iranian (B1B2), non-Iranian domestic (DC), and wild Bactrian camels (WC).

Comparison of identified SNPs for six Bactrian camels revealed that 2,685,723, 1,817,795, 2,586,654, and 800,530 SNPs are common between Iranian, non-Iranian domestic, wild Bactrian camels, and among all samples, respectively (Figure 2(a)). Of shared SNPs among all camels, 406,883 and 393,647 were heterozygote and homozygote, respectively. The 414,436, 687,696, and 108,321 unique SNPs were observed for Iranian, wild, and Mongolia domestic Bactrian camels, respectively. Also, we found 73,953 common INDELs across six individuals (Figure 2(b)).

### 3.3. Annotation of SNPs

**Figure 3:**
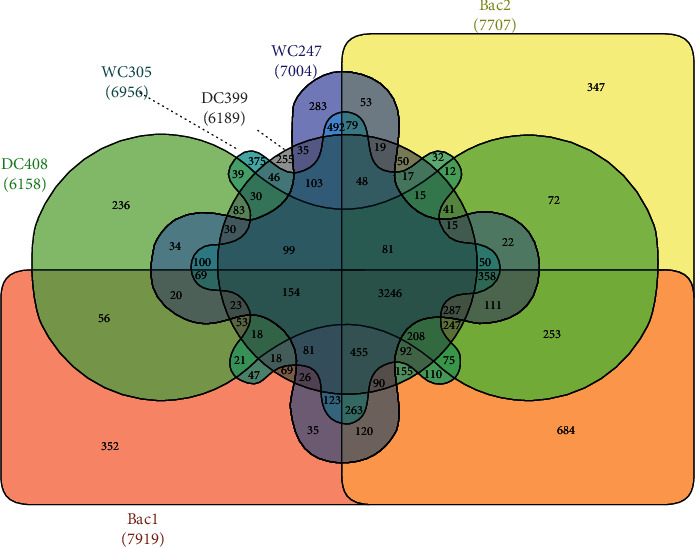
Venn diagram created by InteractiVenn [19], showing the overlap of genes containing missense variants in all studied Bactrian camel.

Annotation of identified SNPs revealed that more than 88.2% of all SNPs are located in intergenic and intron regions (Table 3). Only 0.008 (for DC408) to 0.01 (for Bac1 and Bac2) percent of SNPs have a high impact (stop lost, stop gained, and start lost variants) on products of genes. The number of missense variants ranged from 14,208 for DC408 to 20,003 for Bac1. We extracted affected genes by missense SNPs in shared SNPs among all individuals (All_ind), only_B1B2, only_DC, and only_WC sets and found 2,428, 1,945, 329, and 1,786 genes for the mentioned sets, respectively (Supplementary 1). Some of the SNPs were categorized into more than one functional classes; therefore, we considered them as multi_annotated SNPs with unknown impact and avoided discussing them anymore. Investigation of all affected genes by missense SNPs revealed that 3,246 genes (Supplementary 1) are common among all individuals. These numbers for individuals ranged from 6,158 for DC408 to 7,919 for Bac1 (Figure 3).

### 3.4. Annotation of INDELs

Detected INDELs were classified into seven regional (e.g., intergenic, intron) and seven functional (e.g., frameshift, conservative inframe insertion) variants, as well as some INDELs considered as multi_annotated variants (Table 4). According to the results for SNPs, most of the INDELs occurred in intergenic and introns, and more than 88.6% were common in all samples. We found that ~0.07 percent (ranged from 248 for DC408 to 371 for Bac1) of the INDELs eventuate to frameshift in related genes. We extracted the affected genes by frameshift variants for all individuals, common genes among Iranian and non-Iranian domestic (112 genes), and also those that were common between wild Bactrian camels (174 genes). Eighty genes containing frameshift variants were common among all studied individuals (Supplementary 2).

### 3.5. GO Enrichment of Affected Genes by Missense SNPs and Frameshift INDELs

GO analysis was performed on affected genes by shared missense SNPs in All_ind, only_B1B2, only_DC, and only_WC sets. Results revealed that significantly enriched terms for molecular function are 5 (ATP binding, ATPase activity, calcium ion binding, microtubule motor activity, and motor activity), 2 (Rho guanyl-nucleotide exchange factor activity and transcriptional activator activity and RNA polymerase II core promoter proximal region sequence-specific binding), and 1 (ATP binding) for All_ind, only_B1B2, and only_WC sets, respectively. We found no significant terms in the biological process for mentioned sets; however, one enriched term in cellular component for All_ind set (myosin complex) was found (Supplementary 1). No significant enriched GO terms were observed in biological process, cellular component, and molecular function for only_DC set.

Also, we found that only one GO term under molecular function (neurotransmitter : sodium symporter activity; GO : 0005328) was significantly enriched (*p* adjusted = 0.031) for 80 common genes among all samples, containing a frameshift variant. Enrichment analysis results for common genes among Iranian and non-Iranian domestic Bactrian camels were similar to the mentioned 80 genes; however, no significantly GO term was found for common genes between wild Bactrian camels.

## 4. Discussion

In the present study, we performed the first whole-genome resequencing on two Iranian Bactrian camels to genome-wide variant identification and detailed analysis of them. Obtained short reads after quality filtration were covered 37.4 and 42.6× of Bac1 and Bac2 genomes, respectively. Previously, estimation on whole-genome sequencing data has shown that 15X and 33X mapped read depths are sufficient to identify almost all homozygous and heterozygous, respectively [20]. Although the coverages of downloaded samples are less than our sequenced camel genomes, but it seems to be enough to compare them with obtained results for Iranian Bactrian camels.

Totally, we identified 4,908,998, 4,485,725, and 4,706,654 SNPs for two Iranian, two non-Iranian domestic, and two wild Bactrian camels, respectively. The lower number of detected SNPs in non-Iranian domestic Bactrian camels (belonged to Mongolia), might be resulted from their higher similarity to reference genome (belonged to Alxa breed, a domestic Bactrian camel distributed in China). The heterozygote to homozygote SNP ratio in DC399 (1.75), DC408 (1.79), and Bac2 (1.82) was close, while this value was 3.77 for Bac1. This result was consistent with our previous work on the Iranian dromedary camel which showed higher heterozygosity in them [15]. Based on the microsatellite data, there is introgression between Bactrian and dromedary camels in Iran [6]; therefore, observing higher heterozygosity in a portion of the Iranian camel's population is not unexpected. Also, based on archaeological evidence, Iran is a possible place of origin for the domestic Bactrian camels [21]. Therefore, another probable reason for the high genetic diversity in Iranian Bactrian camels with a small population size [22] can be attributed to the mentioned issue.

Despite the high number of detected SNPs in wild Bactrian camels (WC247 and WC305), heterozygote SNPs in these samples were the lowest in comparison with four domestic camels, so that 52.2 and 46.1% of SNPs were heterozygote in WC247 and WC305, respectively. In many cases, wild species have higher genetic diversity compared to their domesticated breeds, such as pig [23] and dog [24]; however, in some cases such as wild Bactrian camels and for some reasons, this fact can be reversed. One of the reasons for the low heterozygosity in wild Bactrian camels can probably result from their small population size. According to available statistics, there are less than 1,000 wild Bactrian camels in the world [25], which are distributed in China and Mongolia [26].

Annotation results revealed that most portion of the discovered variants throughout the genome of Bactrian camels (more than 88 percent) are located in the intergenic and intronic regions. Our results were consistent with these values for Iranian (87.7% for SNPs, 88.9% for INDELs) and African dromedary camels (88% for SNPs, 89.1% for INDELs; [15]).

The distribution pattern of SNPs in the different regions for common SNPs set was similar to all identified SNPs, so that approximately 89% of shared SNPs among samples were classified as intergenic and intronic variants, and only 0.5% (4,046) of them as missense variants led to amino acid changes (Supplementary 1). The eighty-eight missense SNPs from this set were found in the 30 solute carrier (SLC) gene superfamily. SLC genes encode membrane-bound transporter proteins to transport substrates such as glucose, amino acids, lipids, cation, anion, and many other essential ingredients for the body [27]. SLC27A4 (FATP4) with a missense SNP was one of the important genes in this list. The expression level of the FATP4 gene in the duodenum is higher in comparison with other organs [28]. Encoded protein by this gene plays an important role in the uptake of fatty acids from the small intestine [29]. Lobo et al. [30] reported a reduction in triacylglycerol deposition, diacylglycerol, and monoacylglycerol levels and also lipid droplet sizes in FATP4 knockdown adipocytes. It seems that FATP4 is a key gene for camels because of the importance of fat metabolism in this species. Also, we found four genes (SLC2A4, SLC2A9, SLC5A4, and SLC37A1) related to glucose metabolism and hemostasis in the above-mentioned list. SLC2A4 (GLUT4) is used by insulin as a tool for glucose uptake; in fact, insulin simulates translocation of GLUT4 to the cell surface and, as a result, to glucose uptake [31]. Evidences showed that decreasing in GLUT4 gene expression is associated with insulin resistance and type 2 diabetes in human [32]. SLC2A9 (GLUT9) is another facilitative glucose transporter with one missense SNP in this study. The expression of this gene in the insulin secreting *β*-cells may reflect its function as a glucose sensor in *β*-cells pancreatic islets [33]; however, another important role of this gene is participation in urate homeostasis [34]. SLC5A4 (SGLT3) and SLC37A1 (G3PP) genes that are highly expressed in the digestive system [28] were another affected gene by missense SNPs that play important roles in the absorption and hemostasis of glucose. Considering that camels do not develop diabetes [26], presumably common missense SNPs in glucose metabolism-related genes found in this study have positive effects on camels in dealing with diabetes. Although this is a theory, and to prove it, we need more studies in this field.

Here, we considered common homozygote SNPs among domestic Bactrian camels as fixed SNPs for them, if these variants were absent in the genome of both wild Bactrian camels. It is likely that human selection after domestication is the cause of the fixation of these alleles in domestic Bactrian camels [15]. According to the mentioned criteria, we found 43,944 SNPs, and 16,201 of them were located in the genic region and affected 3,405 genes (Supplementary 1). One of the significantly enriched biological process terms for fixed SNPs set was intracellular signal transduction (GO : 0035556; Supplementary 1), which can be defined as a chain of reactions to transmit chemical signals from the cell surface to their intracellular targets. One of the main targets of these signaling reactions could be transcription factors that ultimately lead to a change in the expression level of genes in response to extracellular stimuli [35].

Investigation in common genes among all individuals that affected by missense variants revealed that 98 of these genes belonged to the zinc finger (ZNF) genes. ZNFs are one member of the large gene family with numerous members and various functions in eukaryotes. Some of the known functions of zinc finger proteins include cell proliferation, differentiation, and apoptosis. Also, zinc finger proteins are involved in skin homeostasis, intestinal epithelium biology, muscle differentiation, adipogenesis, and regulation of cellular stemness [36]. Another interesting issue was the existence of four fertility-related genes (ZP1, ZP2, and ZP4 for females and ZPBP for males) in the list of common genes. The zona pellucida (ZP) is a glycoprotein layer that is synthesized and secreted by mammalian oocytes and surrounds it [37]. ZP in species like human [38], hamster [39], and cat [40] is formed by four glycoproteins (ZP1, ZP2, ZP3, and ZP4) and plays important roles in species-specific sperm-egg binding, preventing polyspermy, ZP-induced acrosome reaction, and protecting the embryo [38]. Zona pellucida binding protein (ZPBP) that is expressed in testis [28] is involved in male fertility, and defects in this gene are associated with abnormal sperm morphology [41]. These results can probably give us some indication about the evolution in the Bactrian camels and by performing more studies in this field, we can better understand the concept of these results. Identification of genetic variation in the whole-genome scale gives us the opportunity of development of approaches like genome-wide association studies and genomic selection [15]. As an achievement, these tools can help in selecting a base population and increasing the number of Iranian Bactrian camels, as well as deepening our knowledge to designing appropriate breeding strategies for this valuable livestock [42].

## Figures and Tables

**Table 1:** Statistics of sequenced reads for Iranian, non-Iranian domestic, and wild Bactrian camels.

Genome	Total reads		Mapped		
	Before trim	After trim	Reads	Bases	Fold coverage
Bac1	813,158,076	797,679,174	775,967,813	74,469,370,345	37.4
Bac2	933,365,664	911,654,292	891,056,256	84,834,523,451	42.6
DC399	638,749,252	638,748,478	631,030,195	59,915,063,832	30.1
DC408	703,381,722	703,381,078	695,555,934	65,880,723,485	33.1
WC247	670,303,728	670,299,044	661,623,617	62,264,294,434	31.2
WC305	669,110,616	669,109,670	660,574,188	62,389,566,081	31.3

**Table 2:** Statistics of sequenced reads for Iranian, non-Iranian domestic, and wild Bactrian camels.

	Bac1	Bac2	DC399	DC408	WC247	WC305
Number of SNPs	3,917,247	3,677,474	3,080,659	3,222,861	3,656,164	3,637,144
TS/TV	2.16	2.14	2.21	2.19	2.26	2.26
Heterozygote SNPs	3,095,932	2,373,043	1,960,836	2,066,909	1,906,913	1,677,539
SNP Het/Hom	3.77	1.82	1.75	1.79	1.09	0.86
Insertion	281,033	262,162	189,259	207,998	224,440	232,150
Deletion	252,155	219,143	169,052	186,214	210,753	217,287
INDEL/SNP	0.14	0.13	0.12	0.12	0.12	0.12

**Table 3:** Functional classification of the identified SNPs for Iranian and non-Iranian domestic and wild Bactrian camels.

SNPs	Bac1	Bac2	DC399	DC408	WC247	WC408
Intergenic	2,157,352	2,026,747	1,686,366	1,778,200	2,000,316	1,992,040
Intron	1,300,679	1,222,106	1,030,347	1,081,521	1,232,401	1,223,725
Upstream gene	214,053	198,502	169,131	170,057	196,542	197,060
Downstream gene	172,846	161,897	141,099	141,095	163,895	162,438
Synonymous	20,755	19,210	15,733	15,013	18,724	18,359
Missense	20,003	19,224	14,741	14,208	16,861	16,529
3′ UTR	16,569	15,358	12,636	12,419	15,186	15,073
nc transcript exon	5,174	5,114	4,369	4,224	4,899	4,715
5′ UTR	4,219	3,928	2,240	2,242	2,601	2,522
nc transcript	669	614	465	430	513	509
Stop gained	203	206	142	144	182	185
Stop lost	148	139	97	100	108	106
Splice region	144	126	85	100	112	110
Start lost	38	36	32	30	33	37
Stop retained	13	15	13	8	20	14
Initiator codon	5	4	2	2	2	3
5′ UTR premature start codon gain	5	7	11	4	9	1
Multi_annotated SNPs	4,372	4,241	3,150	3,064	3,760	3,718

**Table 4:** Functional classification of the identified INDELs for Iranian and non-Iranian domestic and wild Bactrian camels.

INDELs	Bac1	Bac2	DC399	DC408	WC247	WC305
Intergenic	287,116	261,173	191,464	212,718	232,991	240,885
Intron	186,048	166,676	126,188	138,707	153,340	158,081
Upstream gene	30,102	26,765	20,054	21,357	24,305	25,249
Downstream gene	25,109	22,464	17,422	18,277	20,845	21,473
3′ UTR	2,253	1,948	1,508	1,527	1,893	1,942
5′ UTR	393	338	195	197	212	209
Frameshift	371	320	253	248	263	279
nc transcript exon	364	330	247	250	279	280
Conservative inframe insertion	191	172	111	101	129	124
Conservative inframe deletion	153	102	98	88	96	86
Disruptive inframe insertion	133	121	86	75	87	84
Disruptive inframe deletion	81	62	56	45	55	43
Splice region	53	50	33	30	30	30
Bidirectional gene fusion	1	0	0	0	0	0
Multi_annotated INDELs	820	784	596	592	668	672

## Data Availability

All short paired-end reads that used in present study were deposited in SRA database at NCBI with accession numbers SRX3763563 and SRX3774562 for Bac1 and also SRX3773669 and SRX3916437 for Bac2.

## Supplementary Materials

Supplementary 1 Supplementary Table 1: Affected genes by shared missense SNPs among all individuals (All_ind), only between Iranian (only_B1B2), only between non-Iranian domestic (only_DC), and only between wild Bactrian camels (only_WC) sets. Supplementary Table 2: Common genes among all individuals (All_ind), only between Iranian (only_B1B2), only between non-319 Iranian domestic (only_DC), and only between wild Bactrian camels (only_WC) sets that were affected by missense SNPs. Supplementary Table 3: Result of GO analysis for affected genes by shared missense SNPs in All_ind, only_B1B2, and only_WC sets. Supplementary Table 4: Functional classification of common SNPs in all Bactrian camels. Supplementary Table 5: Affected genes by fixed SNPs for domestic Bactrian camels. Supplementary Table 6: Result of GO analysis for genes containing fixed SNPs in domestic Bactrian camels.

Supplementary 2 Supplementary Figure 1: Picture of the Iranian Bactrian camel. Supplementary Figure 2: Fast QC output for the first lane of Bac1 reads, before (A) and after (B) of trimming. Supplementary Figure 3: Venn diagram showing the overlap of common genes among all under study Bactrian camels that were affected by frameshift INDELs.
