# Supplementary material for: Genome-Wide Identification and Analysis of Variants in Domestic and Wild Bactrian Camels Using Whole-Genome Sequencing Data
Source: Int J Genomics. 2020 Jul 15;2020:2430846. doi: 10.1155/2020/2430846 (PMC7381958; doi:10.1155/2020/2430846)
Supplement: Supplementary 2 — Supplementary Figure 1: Picture of the Iranian Bactrian camel. Supplementary Figure 2: Fast QC output for the first lane of Bac1 reads, before (A) and after (B) of trimming. Supplementary Figure 3: Venn diagram showing the overlap of common genes among all under study Bactrian camels that were affected by frameshift INDELs. [file 2430846.f2.docx]

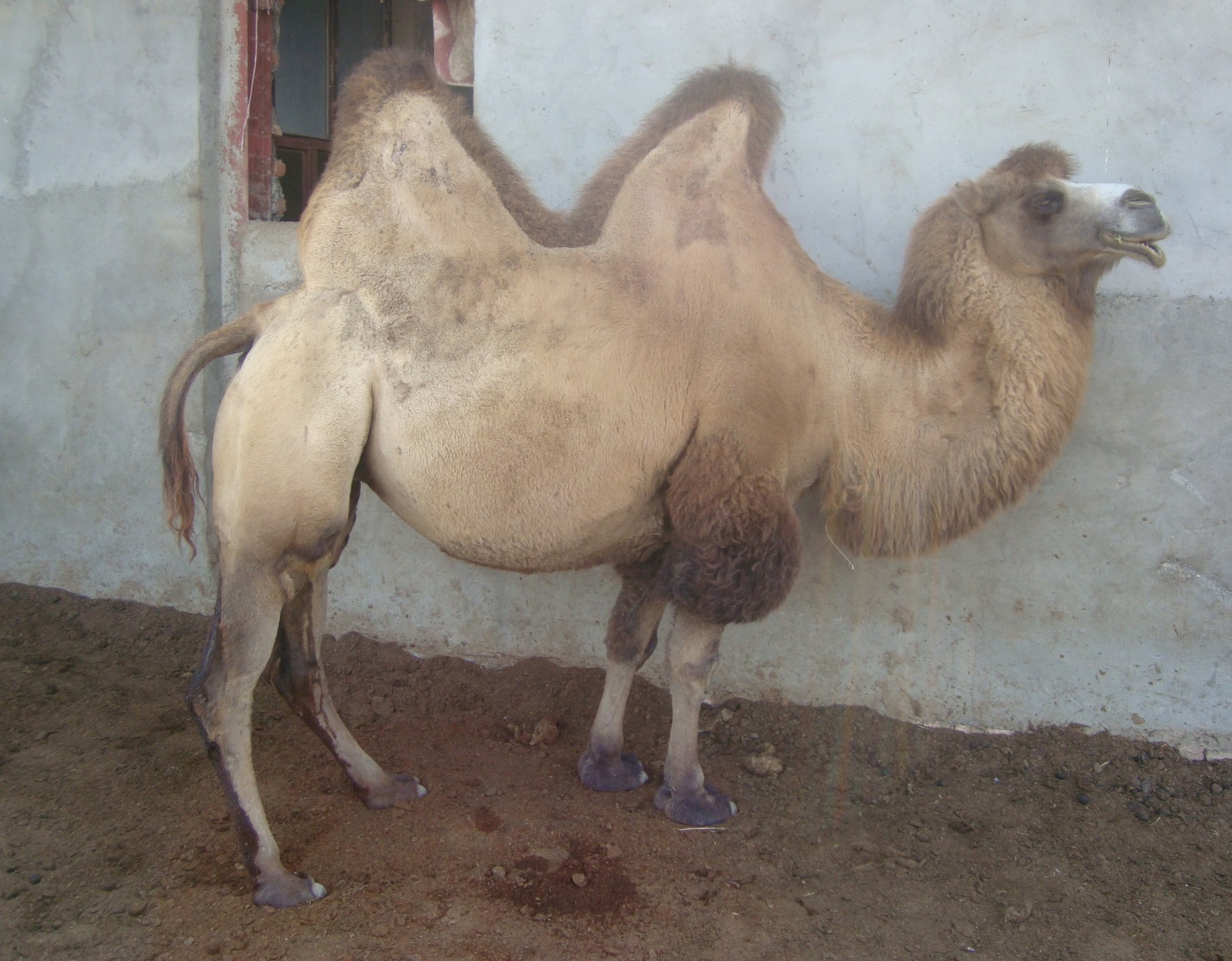
 **Supplementary Figure 1:** Picture of the Iranian Bactrian camel


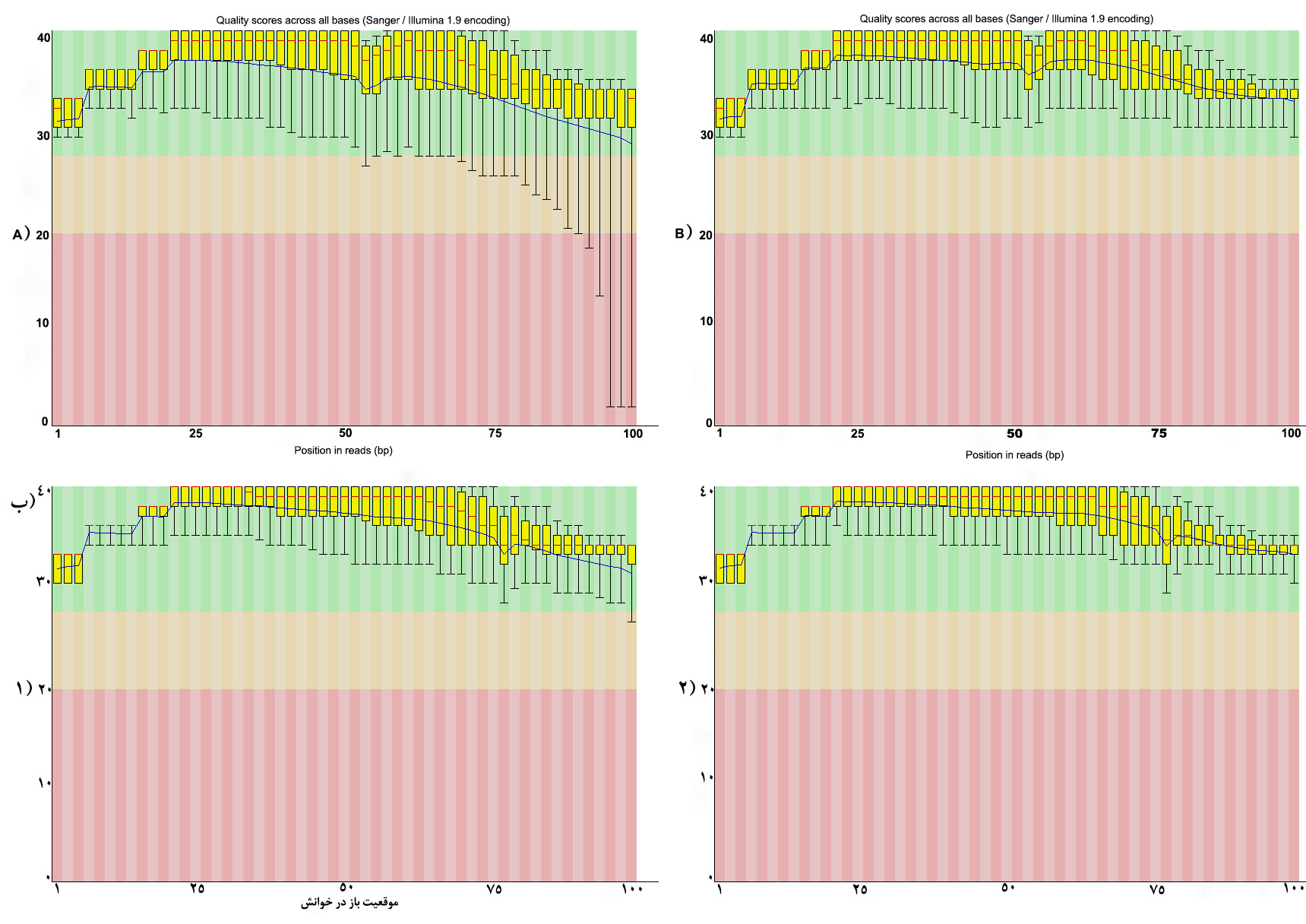


**Supplementary Figure 2:** FastQC output for first lane of Bac1 reads, before (A) and after (B) of trimming


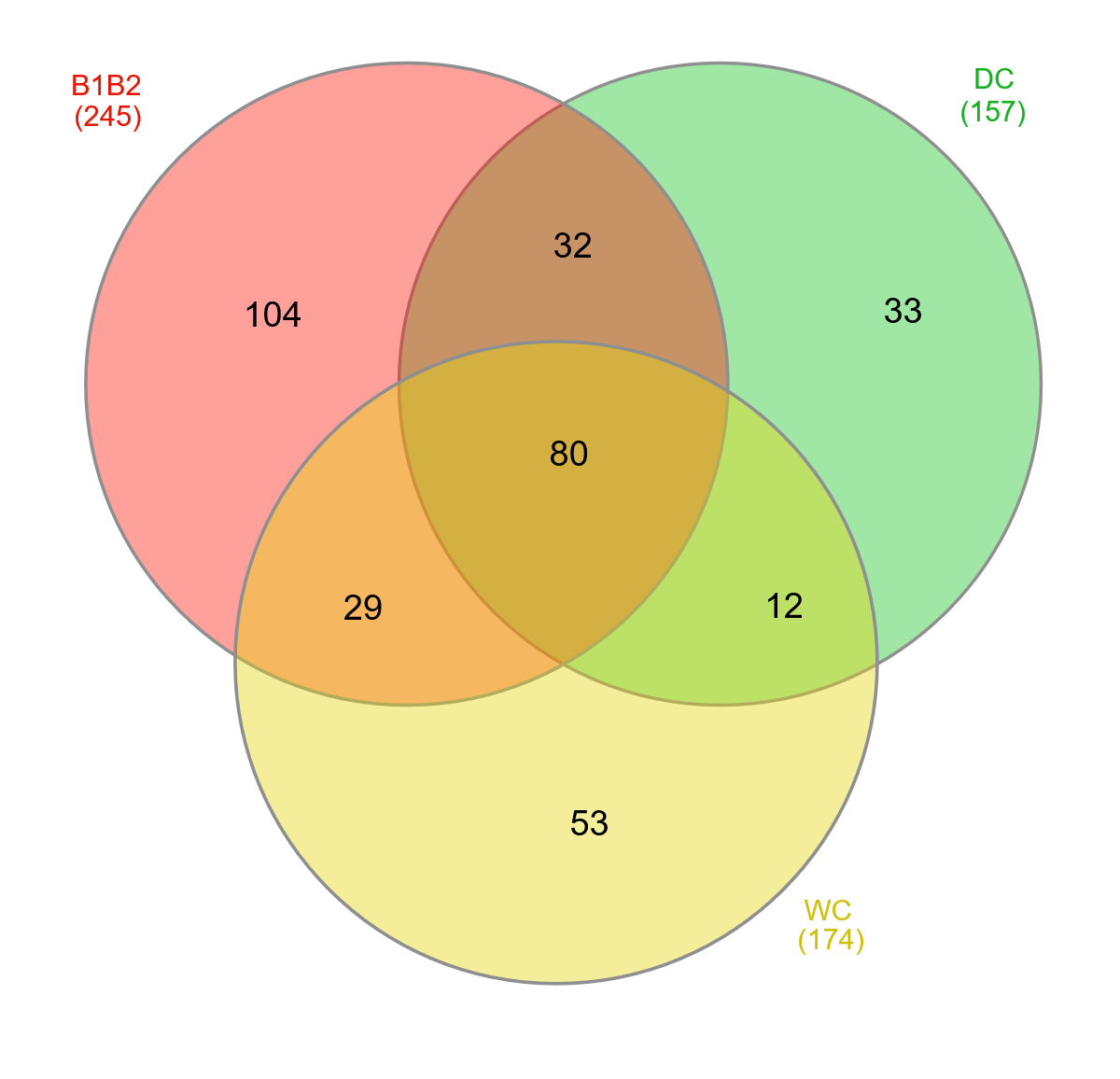


**Supplementary Figure 3:** Venn diagram showing the overlap of common genes among all under study Bactrian camels that affected by frameshift INDELs
